# Selective response of the nucleus taeniae of the amygdala to a naturalistic social stimulus in visually naive domestic chicks

**DOI:** 10.1038/s41598-019-46322-5

**Published:** 2019-07-08

**Authors:** Uwe Mayer, Orsola Rosa-Salva, Jasmine L. Loveland, Giorgio Vallortigara

**Affiliations:** 1 Center for Mind/Brain Sciences, University of Trento, Rovereto, (TN) Italy; 2 Max Planck Institute for Ornithology, Seewiesen, Germany

**Keywords:** Neural circuits, Neuroscience, Social behaviour

## Abstract

The detection of animate beings at the onset of life is important for phylogenetically distant species, such as birds and primates. Naïve chicks preferentially approach a stimulus resembling a conspecific (a stuffed fowl) over a less naturalistic one (a scrambled version of the stuffed fowl, presenting the same low-level visual features as the fowl in an unnatural configuration). The neuronal mechanisms underlying this behavior are mostly unknown. However, it has been hypothesized that innate social predispositions may involve subpallial brain areas including the amygdala. Here we asked whether a stuffed hen would activate areas of the arcopallium/amygdala complex, in particular the nucleus taeniae of the amygdala (TnA) or septum. We measured brain activity by visualizing the immediate early gene product c-Fos. After exposure to the hen, TnA showed higher density of c-Fos expressing neurons, compared to chicks that were exposed to the scrambled stimulus. A similar trend was present in the lower portion of the arcopallium, but not in the upper portion of the arcopallium or in the septum. This demonstrates that at birth the TnA is already engaged in responses to social visual stimuli, suggesting an important role for this nucleus in the early ontogenetic development of social behavior.

## Introduction

One of the earliest expressions of social behavior in newborn vertebrates is a preference to attend to visual stimuli that resemble conspecifics, including stimuli with face-like configurations or biological motion. Interestingly, this phenomenon known as ‘social predispositions’ has been described in humans, non-human primates and domestic chickens alike^1^. These social predispositions emerge in the absence of any specific learning experience, that is, they are observed in visually naïve animals, and have been argued to guide learning towards conspecifics which is important for the subsequent development of neural mechanisms specialized in the processing of social information^2^. This is particularly true for nidifugous birds^3^, such as domestic chickens (*Gallus gallus*), which learn by exposure the visual appearance of their parents, who they will later on follow^4,5^. This form of learning is known as filial imprinting^6–8^. Importantly, key evidence that the configuration of features characterizing the ‘canonical hen’ is crucial to directing chicks’ choice was presented with the finding that chicks prefer a stuffed jungle fowl (the wild ancestor of domestic chickens) over a ‘texture-fowl’ (i.e., small pieces of a stuffed jungle fowl affixed in a scrambled fashion to the sides of a box)^9^. Specifically, the configuration of features contained in the head and neck of the hen seems to be particularly important^9^, and a preference can be found even for schematic face-like stimuli, a simplified representation of the internal structure of a vertebrate face^10–12^. Moreover, visually naïve chicks are also attracted to dynamic features typically associated with the motion of living beings, such as spontaneous speed changes and self-propulsion, which reveal the presence of an internal energy source and to semi-rigid biological motion^13,14^. However, despite the amount of work done on the behavioral characterization of social predispositions, very little is known about the neural mechanisms that are subtended by it (reviewed in^15^).

We conducted a series of experiments to address this issue in visually naïve domestic chicks (i.e., hatched in dark incubators and tested directly after birth) where we mapped neuronal activity through immunohistochemical detection of the expression of the immediate early gene (IEG) c-Fos. The expression of IEGs is tightly linked to neuronal activity and visualizing c-Fos expression in brain sections offers an effective approach to studying neuronal activation in specific brain areas, a technique commonly applied to mammals and birds^16–23^. In our previous studies we found that exposure to the static configuration of features or to the natural motion typical of conspecifics resulted in differential activation in an area involved in filial imprinting (the intermediate medial mesopallium, IMM), as well as in the arcopallium/amygdala complex (a term that has been introduced by Herold *et al*.^24^, to describe a region that combines arcopallium and TnA) and in septal and preoptic nuclei^25–28^. The latter three areas are part of the so-called ‘social behavior network’, which is shared among all vertebrates and comprises interconnected areas rich in sex steroid receptors and implicated in adult social behaviors^29–31^. In our first study, the simple exposure of visually naive chicks to an alive, behaving conspecific, selectively activated septal and arcopallium/amygdala complex including the nucleus taeniae of the amygdala (TnA), revealing their involvement in responses to social companions directly after birth^27^. In follow-up studies, we focused on the role of cues associated with biological, animate motion, and controlled for any effect of static features of the stimuli. We found that both the septum and preoptic area were highly sensitive to the natural motion of a living conspecific^28^ as well as to the movement of an artificial object that simulates self-propulsion on a simplified screen display^25^. On the other hand, the activation observed in arcopallium and nucleus taeniae of the amygdala did not show any differences between the groups exposed to different motion cues.

Taken together these results strongly suggest that the arcopallium and nucleus taeniae of the amygdala might not be involved in the processing of motion features typical of animate creatures. Thus, the activation of these brain areas, which we observed in our original study after the exposure to a living conspecific (another chick), could have been rather due to the static features of this stimulus, such as the appearance of its face region, to which chicks are notoriously responsive. This is particularly intriguing, since at least the nucleus taeniae of the amygdala is considered to be in part homologue to the mammalian amygdala^24,32–37^, which has been implicated in early responses to faces and face-like stimuli in mammals^2,15^.

The arcopallium in birds is a large heterogeneous brain structure, for which multiple subdivisions have been proposed^24,34^. Arcopallial subregions receive input from auditory^38^, trigeminal^39^, somatosensory^40^ and visual pallial areas^41^. The homologies of this region to the mammalian counterparts are not fully understood. While some authors stress its somatomotor component, others believe that all arcopallial regions belong to the amygdala^24,32–37,42–44^. In the most recent paper on this issue by Herold *et al*.^24^, it has been suggested that diverse arcopallial sub regions should be differentiated from the limbic structures (the nucleus posterioris amygdalopallii pars basalis, PoAb and pars compacta, PoAc; arcopallium posterius, AP; arcopallium mediale, AM) which show more similarities with the nucleus taeniae of the amygdala (TnA), a limbic structure located beneath arcopallium. Most of the arcopallium is considered pallial in its nature, whereas TnA corresponds mostly to the subpallial medial amygdala of mammals^32,34–36,45,46^. Like its mammalian counterpart, this area shows an enrichment in androgen and estrogen receptors^47–49^ and is functionally associated with a variety of social behaviors, such as sexual behaviors and social interactions at large^45,50–52^. In fact, the TnA is usually considered to belong to the ‘social behavior network’^29–31^, since it corresponds to the mammalian medial amygdala^32,35,36^. Moreover, in an altricial species, the zebra finch, the TnA can already be delineated at post-hatching day one^51^, suggesting that TnA has the potential to be fully functional during social interactions already at the time of hatching or soon after.

**Figure 1:**
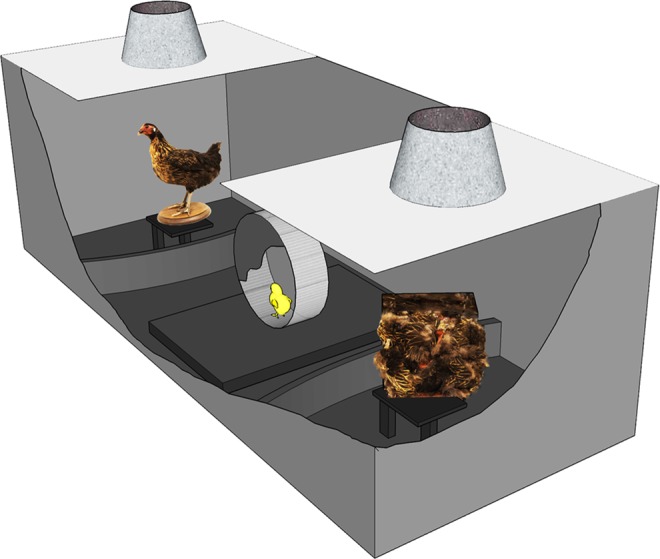
Schematic representation of the experimental apparatus. Visually naïve domestic chicks were placed in the running wheel and were free to run for 30 min towards one or the other stimuli, which were constantly rotating at the ends of the runway. The ‘stuffed fowl’ resembled the static configurations of a conspecific and the control stimulus was a ‘texture fowl’ (i.e., small pieces of an identical stuffed jungle fowl affixed in a scrambled fashion to the sides of a box). The behavior was monitored from above through a video camera and the sum of rotations in one or the other direction was recorded by an automated system.

Since the mammalian amygdala has been implicated in responses to static social stimuli recognizable by specific configurations of features, such as faces^2,53^, in the current study we wanted to investigate if the TnA shows a greater response to a social stimulus that has a face-like structure (i.e. stuffed hen), than to a control ‘texture-fowl’ stimulus that has the same colors and texture but features are arranged in a scrambled fashion. If this prediction were correct, it would reveal a sensitivity of this area to the spatial configuration of features that are typical of most living beings and that elicit inborn social predispositions in naïve chicks. In a first experiment, we thus wanted to confirm, at the behavioral level, the presence of an inborn preference in newly hatched chicks to approach the ‘canonical’ version of a stuffed hen over the ‘texture-fowl’ stimulus. The preference test was performed in a black rectangular arena (Fig. 1) with a running wheel at the center. From inside the running wheel visually naïve chicks could see on one side the ‘stuffed fowl’ and on the other end the “texture fowl”. The “texture fowl” was designed specifically to match the ‘stuffed fowl’ in terms of color, texture and local features, but these features were arranged in a scrambled fashion so as to disrupt any naturally occurring configuration. In the second experiment, we tested whether exposing visually naïve chicks to the ‘stuffed hen’ stimulus resulted in greater c-Fos expression in the nucleus taeniae of the amygdala, compared to the condition of exposure to the control ‘texture-fowl’ stimulus. In addition, we examined c-Fos expression in the arcopallium and in the septum, which is another node of the social behavior network that our previous studies implicated in the response to motion cues of animacy^25,27,28^. We hypothesized that inside the arcopallium/amygdala complex the nucleus taeniae of the amygdala should show stronger activation for the stuffed hen stimulus because it has a face-like configuration in its head region. However, we did not necessarily expect a similar effect in the septum, since our previous studies suggested that the septum is involved mostly in the response to dynamic cues of animacy^25,28^.

## Results

### Experiment 1

All chicks were active in the wheel and made on average 84.2 ± 17.9 (mean ± s.e.m.) total rotations within the 30 min test. Furthermore, all nine chicks ran more towards the ‘stuffed fowl’ with a minimum preference score of 0.51 and maximum 0.86. The average preference score of 0.64 ± 0.04 was significantly different from chance level: t(8) = 3.5099, p = 0.008. The results of this first experiment confirmed that visually naïve chicks prefer the ‘stuffed fowl’ over the ‘texture fowl’. These two stimuli were then used in a second experiment to study the neural correlates underlying this specific case of social predisposition for the stuffed hen.

### Experiment 2

**Figure 2:**
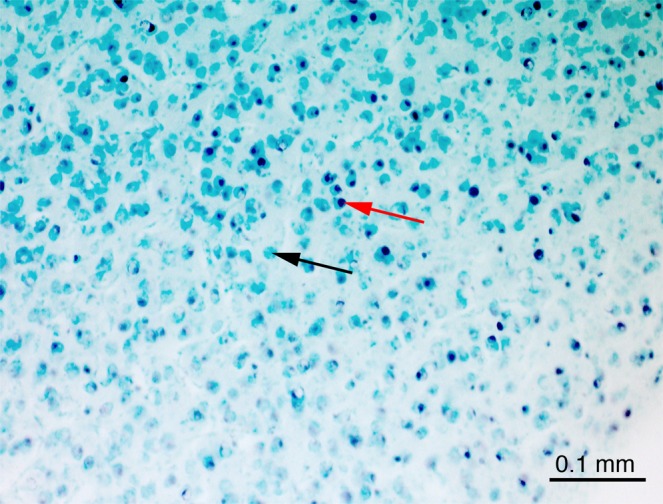
An example of a coronal section showing the region of the nucleus taeniae of the amygdala (TnA) of an experimental chick. c-Fos-positive cells are stained black after the immunohistochemical procedure (red arrow) and are easily distinguishable from the c-Fos-negative cells (black arrow), which were counter stained with methyl-green.

**Figure 3:**
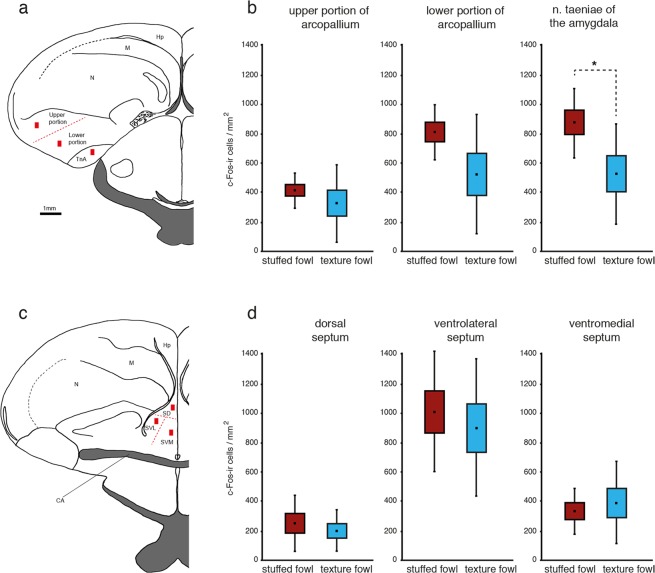
Results of the brain analysis. (**a**) Schematic view of a coronal section showing a typical placement of the cell count zone (red rectangles) within the upper portion of the arcopallium; lower portion of the arcopallium and nucleus taeniae of the amygdala (TnA). (**b**) Significantly higher number of c-Fos-ir neurons is present in the TnA of the experimental chicks compared to controls (n = 8 in each condition). A similar trend is also visible in the lower portion of the arcopallium. (**c**) Typical placement of the cell count zone within the septum with its portioning into dorsal (SD), ventrolateral (SVL) and ventromedial (SVM) subdivisions (red lines). (**d**) The densities of c-Fos-ir cells in any of the septal subregions were not different between the two groups. Graph-plot: mean (black square), standard error of the mean (box) and standard deviation (whisker) (*P < 0.05). Drawings were adapted from the atlas of Kuenzel & Masson (1988). Hp = hippocampus, M = mesopallium, N = nidopallium, Str = striatum, CA = anterior commissure.

In this experiment brain activity in the nucleus taeniae of the amygdala, the arcopallium and septum was compared in chicks after single exposure to the ‘stuffed fowl’ or to the ‘texture fowl’. Chicks exposed exclusively to the ‘stuffed fowl’ made on average 90.6 ± 27 (mean ± s.e.m.) total rotations and the group that was exposed exclusively to the ‘texture fowl’ made on average 53.9 ± 14.7 total rotations, with no significant differences between the two groups: t_(14)_ = 1.1930; p = 0.252. All the brains from the ‘stuffed fowl’ (n = 8) and ‘texture fowl’ (n = 8) groups were successfully processed and stained. The brain sections contained high numbers of c-Fos immunoreactive (c-Fos-ir) cells, which appeared black after the immunohistochemical reaction. Background staining was minimal, thus the c-Fos-ir cells were easily distinguishable from the non-activated cells, which were stained light green due to the methyl-green counterstaining (Fig. 2). We quantified the density of c-Fos-ir neurons in two subdivisions of arcopallium (upper and lower portions of arcopallium), TnA and the three subdivisions of septum (dorsal, ventrolateral, ventromedial), see Fig. 3. Repeated measurements ANOVA revealed a significant interaction of Brain Area*Group: F_(2.367;33.131)_ = 3.149, p = 0.048 (Greenhouse-Geisser correction was applied, because Mauchly test revealed a significant violation of sphericity, p = 0.004), which means that exposure to the ‘stuffed fowl’ or to the ‘texture fowl’ had a different effect on the density of activated c-Fos-ir cells in the different brain areas. Post hoc t-tests revealed significant difference between the two groups in the TnA (t_(14)_ = 2.511; p = 0.025) as well as a non-significant trend in the same direction in the lower portion of arcopallium (t_(8.990)_ = 2.511; p = 0.069, equal variances not assumed based on the results of the Levene test). All the other measured brain regions did not show any differences between the groups (upper portion of arcopallium: t_(14)_ = 1.186, p = 0.255; dorsal septum: t_(14)_ = 0.775, p = 0.613; ventrolateral septum: t_(14)_ = 0.560, p = 0.584; ventromedial septum: t_(14)_ = −0.228, p = 0.823). The raw values (mean ± s.e.m) of the measured c-Fos-ir densities for the two groups in each area were as follows: TnA ‘stuffed fowl’: 877 ± 84, ‘texture fowl’: 529 ± 122; lower portion of arcopallium ‘stuffed fowl’: 813 ± 68, ‘texture fowl’: 525 ± 145; upper portion of arcopallium ‘stuffed fowl’: 416 ± 43, ‘texture fowl’: 328 ± 94; dorsal septum ‘stuffed fowl’: 253 ± 69, ‘texture fowl’: 201 ± 50; ventrolateral septum ‘stuffed fowl’: 1012 ± 146, ‘texture fowl’: 903 ± 165; ventromedial septum ‘stuffed fowl’: 335 ± 57, ‘texture fowl’: 391 ± 100. To check for potential effects of motoric activity we also performed correlation analyses between the total rotations in the wheel and c-Fos-ir densities, for each measured area, considering the two groups both independently and together. None of the correlation tests revealed any significant results. For brevity here we report only the results of the correlations tests for the TnA (Pearson correlation test for ‘stuffed fowl’ group: r = 0.1566, p = 0.711; ‘texture fowl’ group: r = 0.242, p = 0.56; both groups pooled: r = 0.286, p = 0.282).

## Discussion

In the present study, the results from the first experiment demonstrate that visually naïve chicks prefer the ‘stuffed fowl’ over the ‘texture fowl’. This confirms previous results obtained by our and other groups^9,26^, indicative of the robustness of this phenomenon and provides the basis for the second experiment wherein we investigated the neural correlates of exposure to each stimulus. The results of the second experiment demonstrate that differences in c-Fos expression between the two groups exposed to the ‘stuffed fowl’ or to the ‘texture fowl’ are region specific, meaning that not all the regions considered responded to the stimuli in the same way. In particular, we found higher activation of the TnA in response to the ‘stuffed fowl’ compared to the ‘texture fowl’, as revealed by the density of c-Fos-ir neurons. A similar non-significant trend was also present in the lower portion of the arcopallium, but not in the upper portion. In contrast, none of the septal subdivisions showed differential activation between the two stimuli (Fig. 3d). Moreover, number of c-Fos-ir cells showed no relationship to motoric activity. In none of the areas there was any correlation between the total rotations in the wheel and the density of c-Fos expression, suggesting that motoric activity had no effect on the perception of the stimulus. Overall our results mean that the TnA and potentially the lower portion of the arcopallium respond to the static configuration of features that differentiate the two stimuli. In fact, properties such as the visual appearance of texture, color, movement and presence of single features were matched between the two stimuli. Altogether, these results suggest that only part of arcopallium/amygdala complex are sensitive to visual configurations that are characteristic of naturalistic stimuli that are innately preferred by chicks.

In our previous studies we found activation of the arcopallium and TnA in chicks exposed to a conspecific, compared to a baseline condition (i.e. exposure to an empty chamber)^27^. However, these nuclei did not show any differential activation in response to a conspecific when it was compared to a stimulus presenting an identical configuration of static features (a taxidermized chick rotating on its axis)^28^. This indicated that arcopallium and TnA did not respond to the differences between the natural motion of an animate creature and the ‘rigid motion’ of an artificial object, suggesting that these brain areas might be rather sensitive instead, to the static configuration of features typical of animate creatures, if anything. However, this could only remain a speculation since it was based on such indirect evidence. For example, the differential response observed in the arcopallium and TnA in our first study^27^ could have been elicited by the presence of any salient visual object, regardless of its animate appearance. In the current study, we demonstrate for the first time that the TnA and potentially the lower portion of the arcopallium, respond selectively to stimuli based on their animate appearance (configurations of static features that are typical of animate creatures and elicit naïve chicks social predispositions). Moreover, consistently with our previous two studies^27,28^ this differential response can be observed already in visually naïve animals, without the need of any previous specific learning experience.

In the present study the greater activation associated with the naturalistic stimulus was in particular localized in the TnA, which has been suggested to be homologous to the subpallial, medial amygdala of mammals^32,34–36,45,46^. Notably the medial amygdala is considered to be part of the ‘social behavior network’, which consists of a set of reciprocally interconnected brain areas, rich in sex steroid receptors and in control of adult social behavior^30^. Therefore, it is not completely surprising that in particular this region shows the strongest response to the social stimulus in our study. The TnA is an anatomically distinguishable structure, recognizable based on cell density and morphology with simple counterstaining, such as the methyl green used in our study. Even though this region has been suggested to be homologue to the subpallial medial amygdala in mammals^34–36^, its precise location and pallial/subpallial components are debated in the literature. The location of this nucleus is defined differently in the chicken atlases by Puelles *et al*.^54^ and Kuenzel and Masson^55^, and this fact was emphasized also by Hanics *et al*.^33^. The nucleus taeniae (ATn) in the atlas by Puelles *et al*.^54^, is adjacent to the ventral amygdalofugal tract (vaf), which is called tractus occipitomesencephalicus (OM) in the atlas by Kuenzel and Masson^55^. In contrast, the nucleus termed TnA in the Kuenzel and Masson atlas^55^ (see also^56^) lies comparably more ventral to the OM. Furthermore, the ATn nucleus localised by Puelles *et al*.^54^ has been defined as a pallial amygdalar homologue^32^. However, we localized TnA based on the Kuenzel and Masson^55^ atlas, following also Reiner and colleagues^34^. Thus our main finding, the selective neuronal response of the TnA to the social stimulus, refers mostly to the subpallial amygdala^35^, which is rich in androgen receptors^57,58^. The same area is recognized as the subpallial medial amygdala (MeAs)^32^ and can therefore be considered part of the social behavior network^29,30^.

Even more complicated are the subdivisional and homology considerations for the arcopallium in the literature. Overall, the arcopallium in birds is a large heterogeneous brain structure containing both pallial and subpallial parts^37,59^, with subdivisions and homologies that are much less clear^34^. Arcopallium has been divided into the anterior arcopallium (AA), dorsal arcopallium (AD), intermediate arcopallium (AI), medial arcopallium (AM) and has been distinguished from the posterior nucleus of pallial amygdala (PoA) and nucleus taeniae of the amygdala (TnA). The AA, AI and AM send efferents to sensory, somatosensory and motor areas, which makes it very different from the mammalian amygdala and has been a source for conflicting hypotheses regarding its amygdaloid or non-amygdaloid origin^34,37,42,44,60–62^. On the other hand, the PoA contains efferents terminating in limbic areas and most authors agree on its amygdaloid origin^34^. Moreover, the boundaries between any limbic and somatic parts of the arcopallium are unclear^34,63,64^. More recently, also further subdivisions have been proposed for pigeons based on radioactive markings of several transmitter receptors, providing a new detailed map of the arcopallium/amygdala complex^24^. This approach supports a segregation of the pigeon’s arcopallium/amygdala complex into the arcopallium anterior (AA), the arcopallium ventrale (AV), the arcopallium dorsale (AD), the arcopallium intermedium (AI), the arcopallium mediale (AM), the arcopallium posterius (AP), the nucleus posterioris amygdalopallii pars basalis (PoAb) and pars compacta (PoAc), the nucleus taeniae amgygdalae (TnA) and the area subpallialis amygdalae (SpA). Within the arcopallium, the AD has been further subdivided into lateral (ADl) and medial (ADm) parts, the AI was divided into dorsal (AId) and ventral (AIv) parts and the AM into magnocellular (AMm) and parvocellular (AMp) regions. Overall according to these authors ﻿PoAb and AM belong to the viscero-limbic network, while AV is involved in auditory-associative processing^24^, which fits also well with the ‘structural connectome’ proposed for the telencephalon of pigeons^65^, in agreement with connection studies in domestic chicks^33^. The other parts composed of AA, AD and AI, more centrally located in the arcopallium, have premotor-associative functions^65^.

In our present study, the distribution of the activated cells was highly variable between individuals and did not appear to follow precise subdivisions. With regards to the localization on the anterior-posterior axis, our measurements of activity in the arcopallium were localized in sections selected from the region containing the TnA, which is more or less in the middle of the anterior-posterior expansion of the arcopallium. The methyl-green counterstaining did not allow us to confidently identify detailed subdivisions inside the arcopallium. Therefore, we divided the arcopallium in three equal parts between the lamina arcopallialis dorsalis and the upper border of TnA. The most dorsal part was labelled as ‘upper portion of arcopallium’ and corresponds mostly to the AD (as defined for chicks^34^) and also to ADl, ADm and Aid^24^. The negative results we have obtained for the dorsal arcopallium are thus in line with the hypothesis that this area is non-limbic (but see^36^).

The remaining two thirds of arcopallium, located ventrally to the AD and above TnA, comprise the sub-region that we called ‘the lower portion of arcopallium’. Thus, in the current study, the relatively large region that we defined as ‘the lower portion of arcopallium’ covers the AI and AM as described in Reiner *et al*.^34^ and corresponds mostly to the AIv, AV, PoAb and parts of AM as described in Herold *et al*.^24^. This ventral region is heterogeneous in its properties. This might explain why we find only a non-significant trend for a differential response to the social stimulus. As mentioned above AA, AD and AI have somatomotor functions^60,62,65^, AV is involved in auditory-associative processing, whereas only the PoAb and AM are considered to belong to the viscero-limbic network^24^.

An alternative explanation may imply differential sensitivity to visual stimuli in the arcopallium/amygdala complex: naturalistic or life-like social stimuli elicit a greater response (i.e. more c-Fos positive cells) than non-naturalistic stimuli. This is in line with a recent report of visual responsive cells, selective at least for colors and shapes recorded from the dorsal and intermediate arcopallium of pigeons^66^. Arcopallium indeed receives inputs from the entopallium^41^, a telencephalic recipient of the tectofugal visual pathway, one of the two visual pathways reaching the telencephalon of birds as in other vertebrates (collothalamic visual pathway). The tectofugal visual pathway in birds is involved, among other things, in local pattern discrimination, and object and shape information processing^67–69^. This pathway, which receives around 80% of retinal inputs, goes from the optic tectum in the mesencephalon, through the nucleus rotundus of the thalamus to the entopallium, providing visual input to the intermediate arcopallium. The intermediate arcopallium in turn, projects on the optic tectum, completing a tecto-tectal loop^70^ that could theoretically mediate mechanisms of preferential attention to visual social stimuli. Interestingly, at least in humans, amygdala and superior colliculus (homolog of the optic tectum) are believed to be involved in early orienting responses towards social stimuli such as face-like configurations^2^. Moreover it is interesting to notice that the entopallium and its afferent regions may correspond to regions of the ventral stream (‘what pathway’) in mammals that is involved in more sophisticated processing of faces, a function which so far has been only poorly understood in birds^71^.

Finally, it is worth noting that the archistriatum (old nomenclature for several regions of the arcopallium/amygdala complex) has been also implicated in fear responses and rewards associated functions, even though only a limited number of studies investigated this aspect in domestic chicks^63,72^. It remains unclear how these functions are related to the response observed in our study, which involves innate preferences for social stimuli. It is worth noting that these social predispositions guide the subsequent imprinting towards appropriate social objects. Even though the memory storage of filial imprinting has been identified in the intermediate medial mesopallium (IMM^7^), areas of the social behavior network might be involved in other functions associated with the complex phenomenon of filial imprinting, e.g. processing the social valence of and bonding to, social stimuli.

## Methods

### Subjects and hatching conditions

Twenty-five domestic chicks (*Gallus gallus domesticus*) of both sexes of the Ross 308 strain were used. Fertilized eggs were obtained from a commercial hatchery (Agricola Berica, Montegalda (VI), Italy) and were hatched in darkness. Hatching took place at a temperature of 37.7 °C, with 60% humidity. To estimate approximate hatching time, each incubator was equipped with an infrared LED lamp and a camera (CCD Board camera 8.47 mm, 1/3″). Photos were captured every 20 min. Approximately 24 h after hatching chicks underwent an acoustic stimulation procedure, to elicit the subsequent expression of the innate preference to approach the stuffed hen (see^15^). Chicks were positioned in individual cardboard compartments (10 × 10 cm), inside a dark incubator (33 °C) equipped with a loudspeaker. All handling and transportation of the chicks occurred in the dark. Non-species-specific sound stimulation was provided using a digitally constructed audio file composed of non-repeating rhythmic segments of music (same as in Mayer *et al*.^26^). The frequency varied from 100 to 12000 Hz and the sound pressure level varied from 50 to 98 dB. The duration of individual fragments varied from 10 to 60 s and the duration of intervals between them varied from 30 to 90 s. Stimulation lasted a total of 180 min (four 45 min sessions with 15 min intervals between sessions). All chicks were tested approximately 48 h after hatching.

The experiments reported here comply with the current Italian and European Community laws for the ethical treatment of animals and the experimental procedures were licensed by the Ministero della Salute, Dipartimento Alimenti, Nutrizione e Sanità Pubblica Veterinaria (permit number 20269/A).

### Testing apparatus

The apparatus (Fig. 1) consisted of a black rectangular arena, 60 × 50 × 150 cm (W × H × L), with a running wheel at the center (8 cm wide and 25 cm in diameter, TSE Systems, Germany). The stimuli were placed on opposite ends of the corridor at a distance of 50 cm from the center on two rotating platforms (20 rpm). We used the same stimuli as in our previous studies^26^. The stimuli resembled a jungle fowl hen and a ‘texture fowl’ (i.e., small pieces of a stuffed jungle fowl affixed in a scrambled fashion to the sides of a box with dimensions 18 × 18 × 9 cm), used also in the classical studies^9^. Stimuli were illuminated from above (40 W warm light that diffused through a semi-transparent white plastic sheet). Additional illumination was provided by top/front lights (25 W warm light), while the rest of the testing room was dark. The left-right placement of the stimuli in the corridor were counterbalanced between subjects.

### Test of the behavioral preference for the stuffed fowl (Experiment 1)

Nine chicks (4 males) choose between the two stimuli (the ‘stuffed fowl’ and the ‘texture fowl’) placed at the two ends of the apparatus. The test duration was 30 min and was recorded with a video camera. The number of rotations of the wheel in each direction was counted by an automated system.

### Test session for c-Fos labelling (Experiment 2)

Sixteen chicks were randomly assigned to one of the two groups (n = 8 in each group, with 4 males per group). They were tested for 30 min in the wheel as described for Experiment 1, with the only difference that now the apparatus contained only one of the two stimuli. Chicks in the first group were exposed to the apparatus containing only the ‘stuffed fowl’ and chicks in the second group were exposed exclusively to the control stimulus, the ‘texture fowl’. At the end of the test, chicks were placed back in their familiar individual compartments in the dark incubator, where they remained until perfusion (70 min after the beginning of the session). Thus, chicks of both groups had no visual experience before the test and they were handled in identical manner. All procedures occurring after stimulus exposure were performed blind to the experimental conditions.

### Immunohistochemistry

We used a standard procedure for immunohistochemical detection of c-Fos protein in free-floating brain sections (see^22,26^). Chicks were overdosed with an intramuscular injection of 0.3 ml of a 1:1 ketamine (10 mg/ml) + xylazine (2 mg/ml) solution and perfused transcardially with cold phosphate-buffered saline (PBS; 0.1 mol, pH = 7.4, 0.9% sodium chloride, 4 °C) and paraformaldehyde (4%PFA in PBS, 4 °C). To ensure that the frontal sections from all brains had the same orientation we proceeded as described in the brain atlas Kuenzel and Masson^55^. The left and the right hemispheres were processed separately. After each hemisphere was embedded in gelatine (7%) containing egg yolk and incubated in a 20% sucrose in 4% PFA/PBS for 48 h at 4 °C and further 48 h in 30%sucrose in 0.4% PFA/PBS. Four series of 40 μm coronal sections that contained the regions of interest (corresponding to one third of the most posterior part of the telencephalon) were cut on a cryostat (Leica CM1850 UV). Only the sections of the first series were used for labelling. The sections were treated with 0.3% hydrogen peroxide in PBS solution for 20 min, 3% normal goat serum (S-1000; Vector Laboratories, Burlingame, CA, USA) in PBS for 30 min at room temperature and anti-c-Fos antibody in PBS solution (1:2000; rabbit polyclonal, K-25, sc-1669, Santa Cruz, CA, USA) for 48 h at 4 °C. The secondary antibody was an anti-rabbit antibody diluted in PBS (1:2000; BA-1000, made in goat; Vector Laboratories), incubated for 60 min at room temperature followed by signal amplification with the ABC kit (Vectastain Elite ABC Kit, PK 6100; Vector Laboratories). After the visualization of c-Fos immunoreactive products with a VIP kit (SK-4600; Vector Laboratories) the sections were mounted on gelatin-coated slides, dried at 50 °C, counterstained with heated methyl green (H-3402; Vector Laboratories) and cover slipped with Eukitt (FLUKA).

### Brain anatomy

A microscope (Zeiss Axio Imager2) was used to examine the brain sections at a magnification of 200x (eyepiece: 10x, objective: 20x with a numerical aperture of 0.5) and a digital camera (Zeiss AxioCam MRc5). Contrast and exposure time of the camera were adjusted to match the image on the screen to the view under the microscope. The purple-black labelled nuclei of the c-Fos-ir cells were easily discerned from the non-activated cells, counterstained in light green (Fig. 2). Counting was performed blind to the experimental conditions on the computer screen with the Zeiss software (ZEN). For this purpose a rectangular counting area of 150 × 250 μm was positioned inside a region of interest over the spots with highest number of immunolabelled cells (Fig. 3a,c). Every activated neuron within the counting rectangle was marked on the screen with the ‘event marker’, which automatically computed the total number of c-Fos-ir neurons.

To measure cell density within the arcopallium and TnA three to five sections of both hemispheres were selected in the region where TnA was present that would correspond to the plates A8.8 to A6.4 of the brain atlas^55^ (note that the atlas coordinates of two-week-old chicks do not represent the real coordinates in 48 h old chicks). Arcopallium was parsed into a upper portion and lower portion (Fig. 3a). In order to obtain a reliable criterion for counting we divided the arcopallium in three equal parts between the lamina arcopallialis dorsalis and the upper border of TnA. The most dorsal part was labelled as ‘upper portion of arcopallium’. The remaining two thirds of arcopallium located ventrally to the ‘upper portion of arcopallium’ and above TnA, comprise the sub-region that we called ‘lower portion of arcopallium’.

For the measurements within the septum five sections of both hemispheres were selected by the shape and anatomical landmarks that would correspond to the plates A8.8 to A7.6 of the brain atlas^55^. The septum of each section was parsed into dorsal, ventrolateral and ventromedial parts. The border between the ventrolateral and medial septum was based on anatomical landmarks that are visible after methyl green counterstain, whereas the dorsal part was defined as the upper half of the septum starting from the ventral border of the lateral ventricle (Fig. 3c). After completing the cell counts, for each animal the cell densities per mm^2^ obtained from the different brain sections and hemispheres were averaged to estimate overall activity in each of the six measured areas (dorsal, ventrolateral and ventromedial parts of septum, TnA, upper and lower portion of arcopallium). These individual bird means were considered overall indicators for the c-Fos-ir densities within each area and were employed for further statistical analysis.

### Statistical analysis

The total number of rotations made towards the ‘stuffed fowl’ and the ‘texture fowl’ was used to calculate the preference score for the stuffed fowl for each individual chick (number of rotations towards ‘stuffed fowl’/total number of rotations). Values of this score could range from 0 to 1, with a value of 1 indicating an exclusive choice for the ‘stuffed fowl’ and a value of 0 indicating preference for the ‘texture fowl’. A one sample two tailed t-test was used to reveal significant departures from chance level (preference score of 0.5).

Region specific differences between the two treatment groups were analyzed with a repeated measurement ANOVA, with a between-subject factor ‘group’ (two levels) and within subject factor brain area (six levels). Because the assumption of normality of residuals was violated in the raw data (Shapiro-Wilk test: W = 0.966, p = 0.014), a square root transformation was applied (W = 0.980, p = 0.164). For post hoc analysis, independent t-tests were carried out. Statistical analysis was performed with SPSS (IBM v.24.0).

## Data Availability

The raw data supporting the conclusions of this manuscript will be made available by the authors, without undue reservation, to any qualified researcher.
